# Forensic Discrimination Potential of Blue, Black, Green, and Red Colored Fountain Pen Inks Commercially Used in Pakistan, by UV/Visible Spectroscopy, Thin Layer Chromatography, and Fourier Transform Infrared Spectroscopy

**DOI:** 10.1155/2019/5980967

**Published:** 2019-01-06

**Authors:** Mehwish Sharif, Madeeha Batool, Sohail Chand, Zahoor Hussain Farooqi, Syed Azhar Ali Shah Tirmazi, Makshoof Athar

**Affiliations:** ^1^Institute of Chemistry, University of the Punjab, Lahore, Pakistan; ^2^College of Statistical and Actuarial Sciences, University of the Punjab, Lahore, Pakistan

## Abstract

Examination and comparison of fountain pen inks are very important in forensic questioned documents examination in developing countries where the chances of fraud are greater in cases of cheques, marriage papers, entry of birth and death, etc. In this study, fountain pen inks of blue, black, green, and red colours that are commercially used in Pakistan have been discriminated by UV-Vis spectroscopy, TLC, and FTIR spectroscopy. We have calculated and compared the results in terms of discriminating power. UV/Visible Spectroscopy of fountain pen inks of different brands showed different composition despite their similar colours. TLC was effectively used to differentiate between the colored components of inks. FTIR results showed that each brand could be distinguished by studying the pattern of their absorption spectra that appeared due to the presence of different functional groups. On the basis of combined results of UV-VIS, TLC, and FTIR, the DP was found from 0.73-0.8 for blue, 0.80-1.0 for black, 0.5-1.0 for green, and 1.0 for red colored fountain pen inks. Overall, this study demonstrated the elevated worth of analysis of fountain pen inks commercially used in Pakistan as the study for fountain pen inks, while not very common, remains an interesting target study.

## 1. Introduction

The fountain pen is being extensively used as an instrument of writing for more than 10 decades. In our public existence, it is regularly utilized to sign official manuscripts. In forensic questioned document examination, criminal cases concerning inaccurate or rewritten documents come across for examination and, therefore, the way for detection and dating of fountain pen ink has a vital role in that field [1]. In most of the cases, the capability to discriminate between different inks can be very helpful. Backdate amendment in a document by writing with a fountain pen containing indistinguishable (the same) color may show the dissimilar composition of dye. Thus, the comparison of inks may establish a connection between two specimens in a case of forgery [2]. However, it is not easy to analyze inks by nondestructive techniques that do not have a problem of spectral contamination due to underlying substrate [3].

Ink applied to the surface of paper contain similar main components of dye and, however, may further be differentiated by differences in their IR spectrum [1]. Fountain pen inks are water-based inks. A good fountain pen ink must be free of sediment, noncorrosive, and free-flowing [4]. Water soluble inks can additionally hold extremely fine pigments and synthetic acid dyes. The study of ink is a great challenge for analysts because of its intricate composition, and, therefore, diverse analytical techniques are used [5]. The recognition as well as assessment of fountain pen inks has been seldom completed [6, 7]. The majority of inks used for fountain pens are formulated by complex combination of chemical compounds together with color imparting products (such as iron (II) sulfate, acid dyes, basic dyes or direct dyes, and inorganic or organic colored pigments) and chemical additives (such as surface active agents, adjusters for viscosity, antioxidants, resins, glycerol, and glycol) [8]. Dye components are sensitive to light as compared to other additives of ink and would disintegrate gradually in the normal atmosphere [9]. Consequently, the examination of the colorants of dye can provide a valuable knowledge for the aging of ink compared to the examination of additives of ink [10, 11].

In order to differentiate between the inks, TLC has become a typical method for routine analysis [5], whilst another technique that can be utilized for more elaborate knowledge regarding the organic composition of the ink is Fourier Transform Infrared (FTIR) Spectroscopy [2, 12, 13]. UV/Visible Spectroscopy discriminates fountain pen inks mostly on the basis of their dyestuff content or color. In recent years, various studies have come up with a variety of analytical methods to discriminate script inks [14]. A number of spectroscopic techniques like inductively coupled plasma atomic emission spectroscopy (ICP-AES) and inductively coupled plasma mass spectrometry (ICP-MS) are not used for routine analysis as they require high maintenance and are very costly. In such circumstances, the analyst has to use combination of techniques which require less sample preparation and are frequently available [15].

More recently, for statistical representation, multivariate analysis has become popular among the researchers for forensic discrimination of samples of paper [16], inks [17–20], toner [21], counterfeit currency [22], determination of age [23], examination of soil [24], analysis of fibers [25], and nuclear forensic [26]. In this study, the sample size (n=5 replica) is too small to run multivariate analysis. Therefore, discriminating power is used in this study.

The purpose of this study is to prepare ink examination datum for fountain pen inks used in Pakistan as the data for fountain pen inks used in Pakistan is not available. Therefore, the study of fountain pen inks is novel. There are methods for the nondestructive examination of inks including Video Spectral Comparator (VSC), Raman Spectroscopy [18, 27–31], and luminescence spectroscopy [32] that can analyze ink samples without affecting the sample's integrity. This study will help questioned document examiners in their forensic casework to distinguish inks that cannot be discriminated by nondestructive techniques and to further authenticate them in the court of law. Forensic analysts have knowledge about the use of these techniques for discrimination of inks [28, 33, 34]; however, an extensive datum regarding discriminating power for the succession of comparative techniques is not found in the literature for fountain pen inks.

## 2. 2. Materials and Methods

### 2.1. Samples Preparation

Total 18 ink bottles/pots of fountain pen inks (blue fountain pen inks of six different brands, black fountain pen inks of six different brands, green fountain pen inks of four different brands, and red fountain pen inks of two different brands as shown in Table 1) commercially used in Pakistan were acquired from local market of Lahore, Pakistan. Each ink was initially applied to a white paper of A4 size and 80 gms (Double-A Premium) using a fountain pen. It was allowed to dry for 20-25 minutes. Paper containing fountain pen ink sample was punched using Harris Aluminium Micro Punch of 1mm and its ink was extracted using distilled water as a solvent in a glass vial. For each sample preparation, 30 micro plugs of paper containing ink were dissolved in100*μ*L distilled water. For reproducibility, five replicate samples of each ink sample were prepared. A control sample comprised of blank paper in distilled water was also prepared to compare the effect of the matrix.

In parallel, fountain pen ink samples were also used directly with dilution with water wherever required to assure the effect of sampling on analytical results.

The discriminating power of techniques was calculated according to the formula available in the literature [35]:

(1) $$\begin{array}{c} DP=\frac{number\;\;of\;\;discriminating\;\;sample\;\;pair}{number\;\;of\;\;possible\;\;sample\;\;pair}. \end{array}$$

#### 2.1.1. UV-Visible Spectroscopy

Samples for UV-Visible spectroscopy were prepared by transferring 10 *μ*L ink extract of each ink in a separate sample vial and diluting it with 3.5 ml distilled water. T90+ UV-VIS spectrophotometer, PG Instruments Limited, Beijing, China, was used for UV-Visible analysis. The results (absorbance) were recorded in the spectral range of 230-800 nm. The data was processed with UV win 5 Spectrophotometer software. A Quartz cell having 1cm path length was used for the measurement of absorbance of all samples.

#### 2.1.2. Thin Layer Chromatography

Each extracted ink sample was spotted on TLC plate (Silica gel 60 F_254_(20 x 20 cm), Merck, Germany) using a capillary tube of 0.5 mm. Different solvent systems were tried/checked for the experiment, e.g., Ethyl Acetate : Ethanol (70 : 30), Ethyl Acetate : Ethanol : Ammonia (70 : 30 : 0.5), Butanol : Ammonia : Water (60 : 15 : 25), and Butanol : Acetic Acid : Water (60 : 15 : 25). Best results were obtained using Butanol, Acetic Acid, and Water (60 : 15 : 25); therefore, all samples of blue (BL1-BL6), black (BK1-BK6), green (GN1-GN4), and red (RD1-RD2) inks were eluted using Butanol : Acetic Acid and Water (60 : 15 : 25) as solvent system. The retention factor (Rf) and color tones of the separated bands were determined. The procedure was repeated five times for each sample to obtain better reproducibility.

#### 2.1.3. FT-IR Spectroscopy

IR analysis was performed using Agilent Cary 630 FTIR instrument using Sample interface Diamond ATR with 32 samples as well as background scans and 2 cm^−1^ spectral resolution; the spectral range used was 4,000 cm^−1^ - 650 cm^−1^ using Agilent MicroLab PC software, Automated IQ/OQ, 21 CFR Part 11 compliant, Resolutions Pro for advanced data analysis.

10 *μ*L of each ink extract was spotted directly onto the diamond ATR accessory. For good absorption of IR, each ink was directly spotted on a diamond crystal, independent of the amount of ink-spotted but enough to cover crystal area.

### 2.2. Software

All graphs are normalized using Origin Software (version 6.0).

## 3. 3. Results and Discussion

### 3.1. UV-Visible Spectroscopy

**Figure 1:**
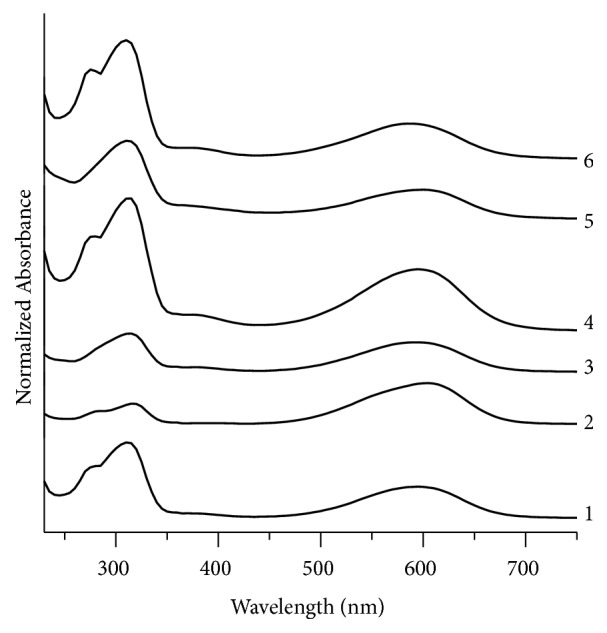
Normalized UV/Visible absorption spectra for blue fountain pen inks. 1, 2, 3, 4, 5, and 6 represent samples no. BL1, BL2, BL3, BL4, BL5, and BL6, respectively.

UV-Visible spectroscopy is a principal tool to investigate coloring agents in inks [36]. The fountain pen ink samples examined in this study have been discriminated on the basis of qualitative analysis of UV/Visible absorption spectra. The discrimination is based on minor differences in peak positions and their relative intensities as shown in Table 2. In blue ink samples BL1, BL2, BL4, and BL6, a prominent absorption band appears at 315 nm with a small shoulder at 280 nm (Figure 1). However, this shoulder does not appear in samples BL3 and BL5. Another band at 595 nm is seen in samples BL1, BL3, and BL4 that is moved to 605 nm, 601 nm, and 585 nm in samples BL2, BL5, and BL6, respectively. Samples BL1, BL3, BL4, and BL6 show a minor peak at 385 nm while this peak is less significant in sample BL1 but BL2 and BL5 have not shown that peak. From the above discussion, it is inferred that blue ink pairs BL1, BL4; BL1, BL6; BL3, BL5; and BL4, BL6 are nondiscriminating pairs.

**Figure 2:**
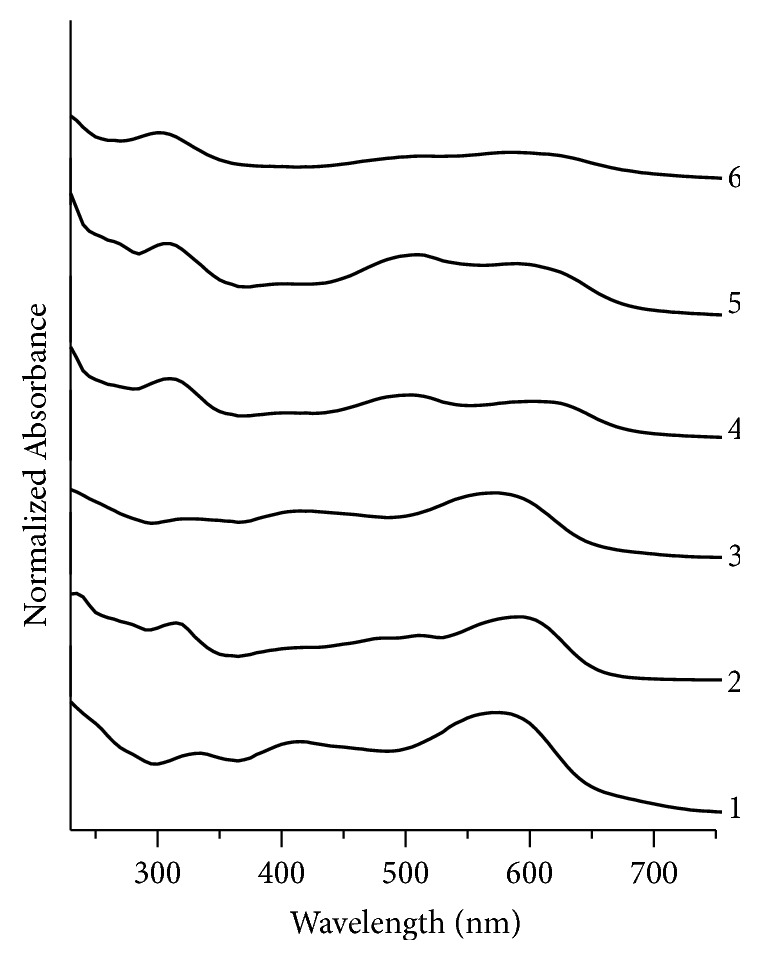
Normalized UV/Visible absorption spectra for black fountain pen inks. 1, 2, 3, 4, 5, and 6 represent samples no. BK1, BK2, BK3, BK4, BK5, and BK6, respectively.

Among black fountain pen inks (Figure 2), sample BK1 shows a large band at 575 nm while this band is slightly shifted in samples BK2, BK3, BK4, BK5, and BK6. Samples BK4, BK5, and BK6 show another absorption band at 500 nm, at 510 nm, and at 507 nm, respectively, which does not appear in the UV spectrum of the rest of the samples. Samples BK1 and BK3 both have shown a small band at 415 nm that is absent in the rest of the samples. All black fountain pen ink samples selected for this research BK1-BK6 show a small band at different wavelengths, i.e., at 335 nm, 315 nm, 320 nm, 310 nm, 305 nm, and 302 nm, respectively. From Figure 2, it is inferred that all sample pairs of black fountain pen inks except BK1, BK3 and BK4, BK5 have been discriminated.

**Figure 3:**
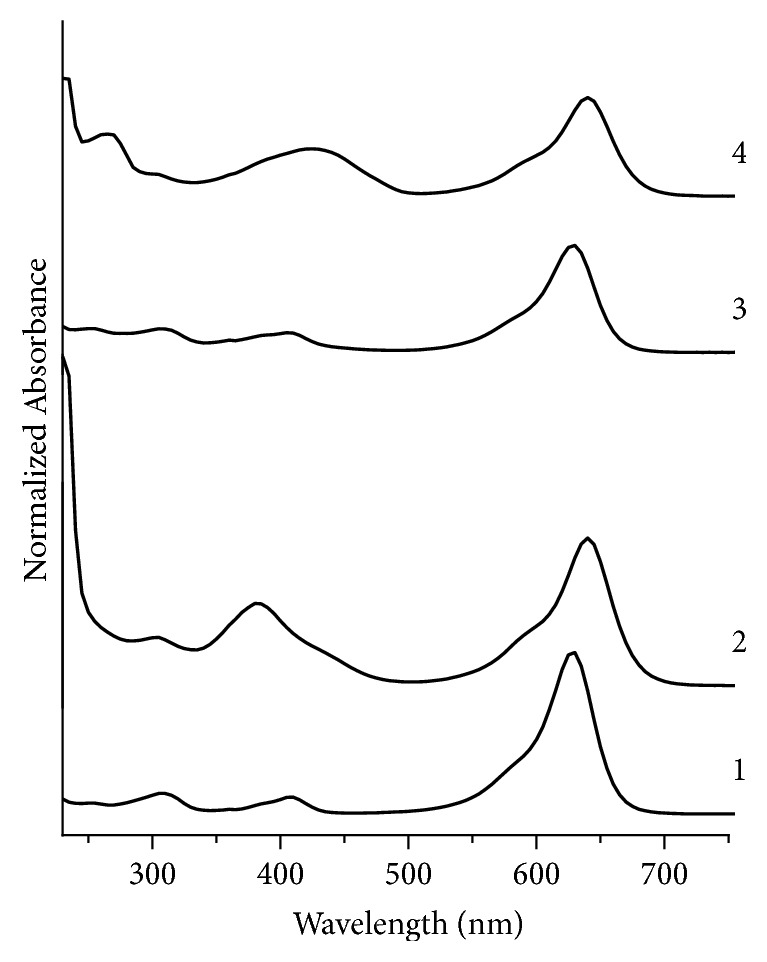
Normalized UV/Visible absorption spectra for green fountain pen inks. 1, 2, 3, and 4 represent samples GN1, GN2, GN3, and GN4, respectively.

In Figure 3, green fountain pen inks GN1 and GN3 show a large band at 630 nm; however, this large band is shifted to 640 nm in the UV-Visible spectra of samples GN2 and GN4. Sample GN2 shows another large band at 385 nm which is shifted to 430 nm in sample GN4; however, in the case of samples GN1 and GN3, this abovementioned band appears at 410 nm. A couple of other bands appear in samples GN1 and GN3, as mentioned in Table 2. In view of the above, it is clear that sample GN1 and sample GN3 are a nondiscriminating pair.

**Figure 4:**
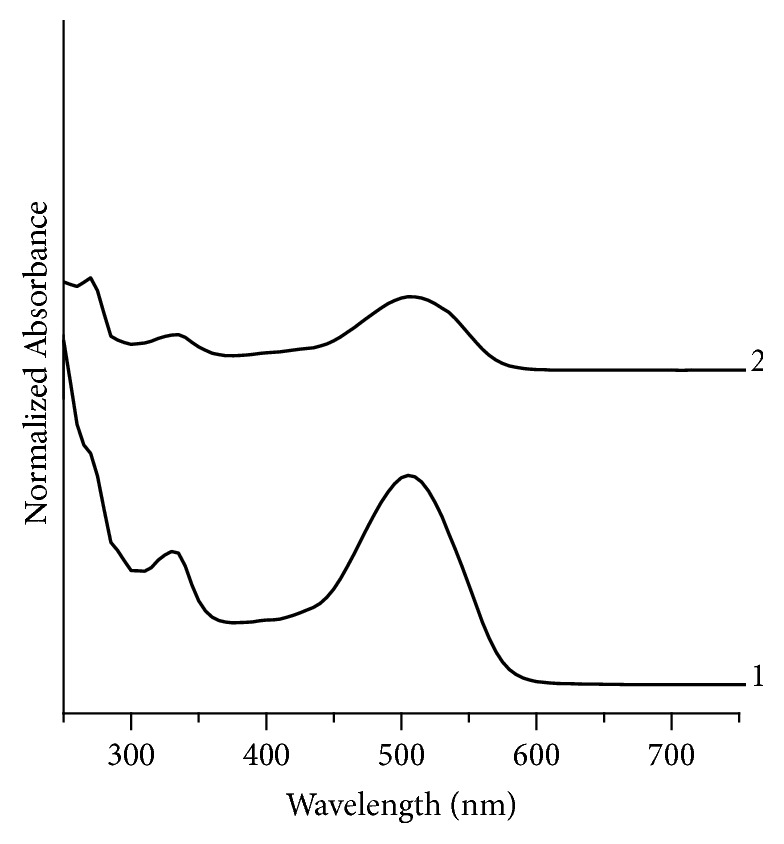
Normalized UV/Visible absorption spectra for red fountain pen inks 1 and 2 represent RD1 and RD2, respectively.

Similarly, comparison of UV-Vis spectra (Figure 4) of red inks shows the appearance of bands at different positions and thus making samples RD1 and RD2 a discriminating pair.

Five replicas of all samples were run to assess the repeatability of the measurements. Similar results were obtained when compared with the samples of inks extracted from the paper in water. In such experiments, the relative standard deviation (RSD) observed was 4%. For interpreting the results, understanding of discriminating power (DP) of an examination is of enormous help to forensic analysts, although a very limited information is present in literature for the discriminating power [37, 38]. In the present research, the discriminating power calculated on the basis of qualitative information is 0.73, 0.87, 0.83, and 1.0 for blue, black, green, and red fountain pen inks, respectively, as calculated in Table 3. The DP of blue pen ink by UV-Vis spectroscopy calculated in this study is slightly higher than for blue pen inks in the literature, 0.71 [39].

### 3.2. Thin Layer Chromatography

TLC is the most extensively used technique for routine analysis because of its simplicity and low cost [33, 34, 40]. In this study, TLC has been used to discriminate fountain pen inks on the basis of qualitative analysis. Inks have been divided into four groups on the basis of their color, i.e., blue, black, green, and red.

**Figure 5:**
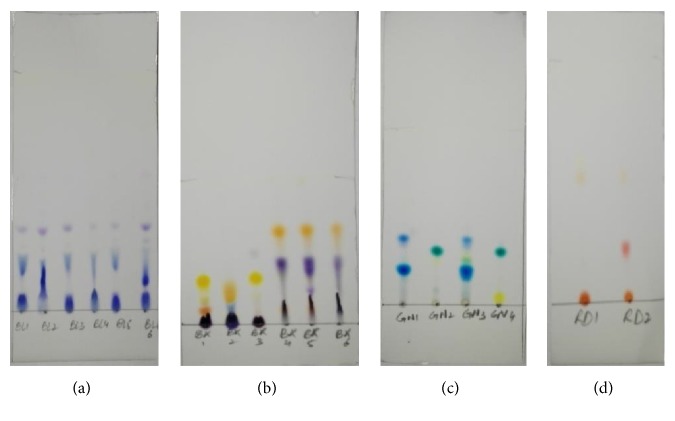
TLC plate developed for (a) blue fountain pen inks, (b) black fountain pen inks, (c) green fountain pen inks, and (d) red fountain pen inks.

Chromatogram of blue ink samples BL1-BL6 (Figure 5) developed by the abovementioned solvent system shows bands of blue, sky blue, and purple colors with different Rf values as mentioned in Table 5. The results show that components of fountain pen inks except for BL1, BL3; BL1, BL5; and BL3, BL5 have been discriminated by TLC. Discriminating power of TLC can be enhanced by adjusting the solvent system.

Among the samples of black fountain pen inks, samples BK1-BK4 show three bands of different colors, whilst samples BK5 and BK6 show four bands and two bands, respectively. From the results mentioned in Table 5, it is clear that all six samples of black fountain pen inks show bands with different Rf values.

For TLC chromatogram of green fountain pen ink samples, the results indicate that samples GN1 and GN2 show three bands of different colors with different Rf values, and sample GN3 shows five bands while sample GN4 shows only two bands of different colors.

Similarly, red fountain pen inks, i.e., samples RD1 and RD2 show a different number of bands of different colors with different Rf values. Sample RD2 shows three bands while sample RD1 shows four bands of different colors.

On the basis of qualitative information using TLC, discriminating power calculated for this study (as mentioned in Table 4) is 0.80 for blue colored inks that are lower than blue pen ink studies in the literature, 0.90 [34] and 0.99 [41]. For black, green, and red colored fountain pen inks, discriminating power calculated in this study is 1.0. These DP values calculated from TLC results have been found better compared to forensic studies available in the literature for black inks, i.e., 0.89 [36], 0.9 [34], 0.95 [41], and 0.99 [12], for red inks, 0.85, and green inks, 0.66, respectively, using TLC [41].

### 3.3. FT-IR Spectroscopy

The discrimination of inks by FTIR spectra is achieved due to the difference in the number of peaks, peak positions, and peaks intensities with respect to each other [3]. An IR spectrum shows two regions, i.e., functional group region (above 1500 cm^−1^) which is used to identify different functional groups present in the ink and fingerprint region (below 1500 cm^−1^) which helps in characterizing the whole molecule. Although the fingerprint region can help in the identification, it does not help in final conclusion [42]. The most important region for ink analysis is 1800-650 cm^−1^ [43]. The IR spectrum of a complex mixture such as ink is an overall representation of the mixture and making any specific compound identification is not possible. In this study, the differentiation of fountain pen ink samples by FTIR spectra has been achieved on the basis of qualitative comparison of absorption bands of different samples. Differences in the intensity of bands were considered valuable only when large and macroscopic changes in the relative intensities of adjacent peaks were found. As in the sampling of fountain pen inks, their age was not controlled, and the intensity ratios used in the aforementioned works [13, 28] were not considered for the discrimination of fountain pen inks. The effect of aging on discriminating power is not considered in the present work.

**Figure 6:**
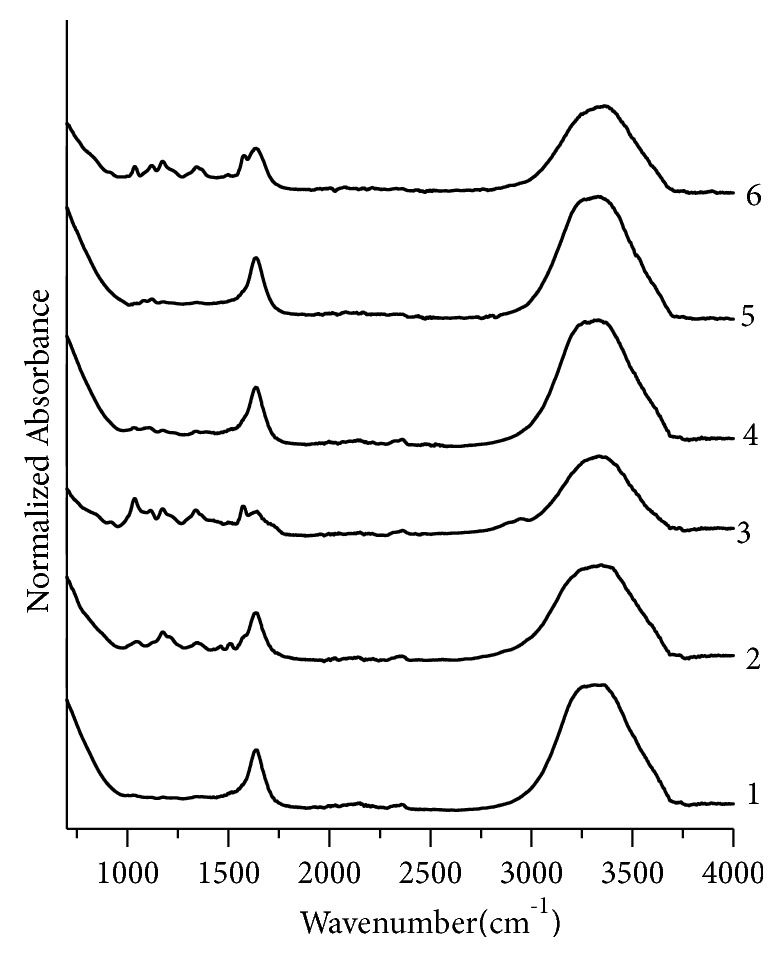
Normalized FTIR absorption spectra for blue fountain pen ink. 1, 2, 3, 4, 5, and 6 represent samples no. BL1, BL2, BL3, BL4, BL5, and BL6, respectively.

Absorption peaks for blue fountain pen inks have been marked by comparing their positions with the literature [44]. FTIR spectra for blue fountain pen ink samples exhibit absorption peaks in the range 3337-3325 cm^−1^, as shown in Figure 6. This band is attributed to O-H group by comparing the results with the literature [44]. Most of blue ink samples show absorption peaks between 1635 and 1632 cm^−1^ due to the presence of N-H group [44], at 1574-1569 cm^−1^ due to the presence of carboxylic acids or its derivatives, carboxylates (salts), amino acid zwitterions [44], and at 1509-1506 cm^−1^ due to the presence of aromatic C=C bending [44]. Fountain pen ink samples show absorption peaks at 1469 cm^−1^ due to C-H bending or C-C stretching (in the ring) [44] and between 1349 and 1335 cm^−1^ due to nitro compounds, N-O symmetric stretch [44]. The peak for ester linkage at 1173 cm^−1^ is common; however, some additional peaks for ester linkage appear in the range 1123-1119 cm^−1^ [44]. IR absorption peaks for fluoroalkanes between 1012 to 1000 cm^−1^ and for aliphatic amines at 1045 cm^−1^ [44] are observed in samples. Fountain pen ink samples exhibit absorption peaks at 918 cm^−1^ and 913 cm^−^1, respectively, that represent organosilicone and phosphorus compounds [44]. All samples pairs except BL1, BL4; BL1, BL5; BL3, BL6; and BL4, BL5 have been discriminated (Figure 6).

**Figure 7:**
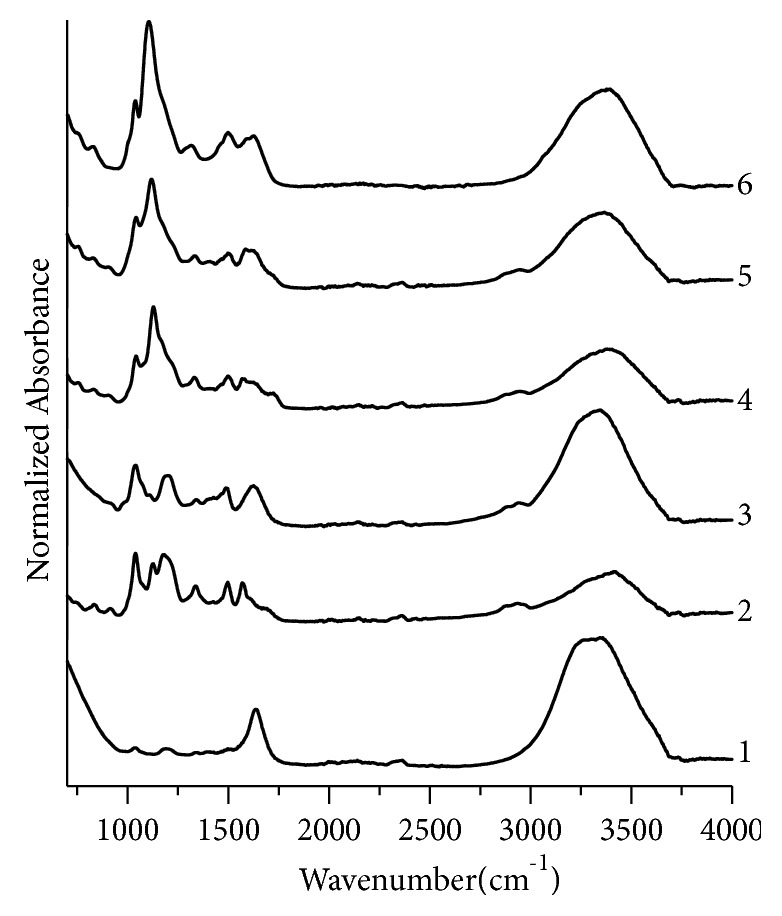
Normalized FTIR absorption spectra for black fountain pen inks. 1, 2, 3, 4, 5, and 6 represent samples no. BK1, BK2, BK3, BK4, BK5, and BK6, respectively.

**Figure 8:**
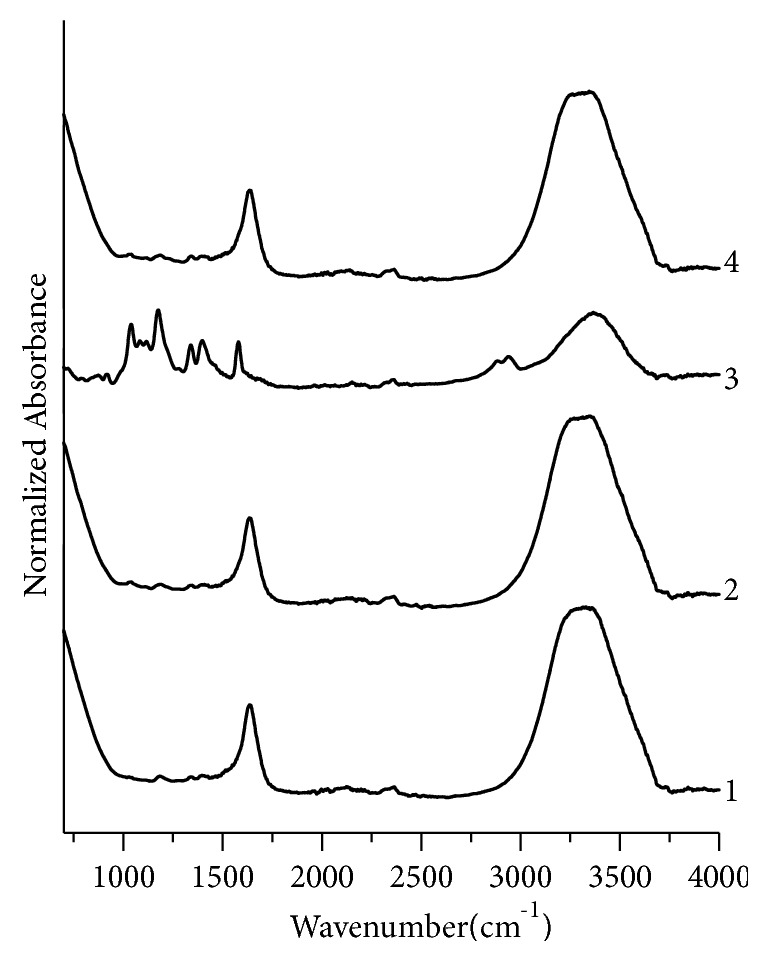
Normalized FTIR absorption spectra for green fountain pen inks. 1, 2, 3, and 4 represent samples no. GN1, GN2, GN3, and GN4, respectively.

**Figure 9:**
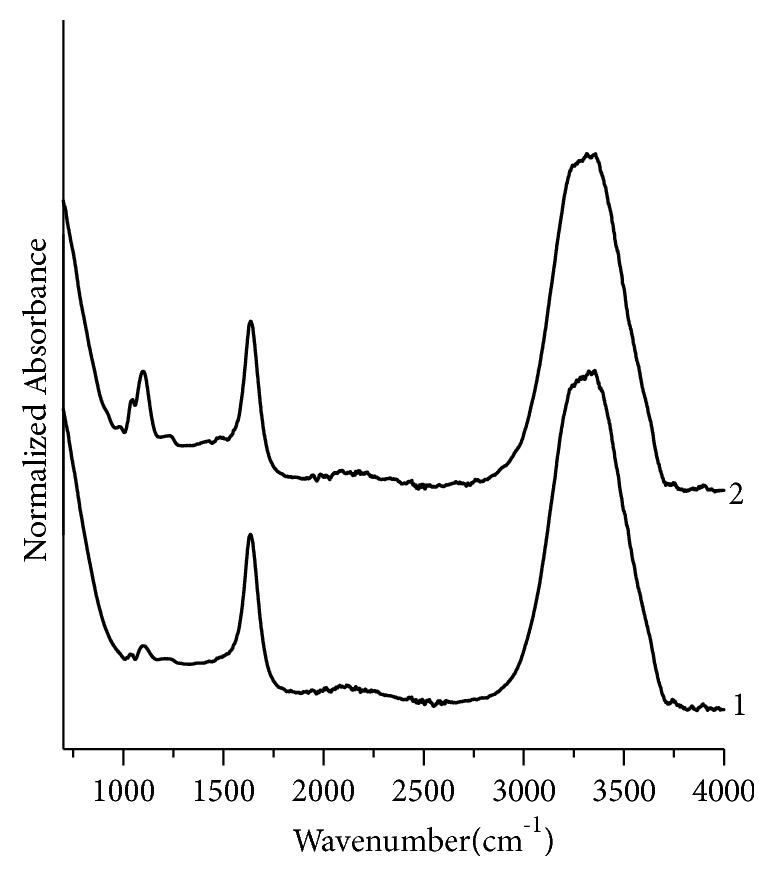
Normalized FTIR absorption spectra for red fountain pen inks. 1 and 2 represent samples no. RD1 and RD2, respectively.

Similarly, the assignment of peaks for black, green, and red fountain pen inks has been made by comparing their IR data with the literature. For black fountain pen inks (Figure 7), three sample pairs BK4,BK5; BK4,BK6; and BK5,BK6 are nondiscriminating pairs. GN1,GN2; GN1,GN4; and GN2,GN4 are nondiscriminating pairs for green fountain pen inks samples (Figure 8) and there is no nondiscriminating pair for red inks (Figure 9).

On the basis of qualitative information, discriminating power for this study by using FTIR has been found as 0.73 for blue, 0.80 for black, 0.5 for green, and 1.0 for red colored fountain pen inks (Table 6).

## 4. Conclusion

The data for forensic discrimination potential of different techniques or for the succession of techniques used for fountain pen inks are not extensively accessible as most of the data available is for ballpoint pen inks. In the present study, the discriminating power (DP) for fountain pen ink was found to be 0.73, 0.87, 0.83, and 1.0 for blue, black, green, and red colored inks, respectively, using UV/Visible Spectroscopy. DP for fountain pen inks was found to be 0.80, 1.0, 1.0, and 1.0 for blue, black, green, and red colored inks, respectively, using Thin Layer Chromatography. DP for fountain pen inks was found to be 0.73, 0.80, 0.5, and 1.0 for blue, black, green, and red colored inks, respectively, using Fourier Transform Infrared Spectroscopy. The discriminating powers, i.e., 0.73 and 0.5, calculated from FTIR analysis of blue and green inks, respectively, show that FTIR for blue and green fountain pen inks is not as good as UV/Visible Spectroscopy and TLC. One pair of blue fountain pen inks BL1 and BL4 was indiscriminating by UV/Visible and FTIR spectroscopy, but by applying TLC it was discriminated. TLC and UV/Visible have been found to be more efficient and reliable techniques for analysis of fountain pen inks as compared to FTIR technique. By using TLC, BL1, BL3, and BL5 cannot be discriminated. The UV-Visible spectra of these 3 inks show a difference in the UV. BL1 has a shoulder at about 280 nm to the peak at about 300 nm. There is no shoulder at this position on BL3 and BL5 indicating that UV/Vis spectroscopy gave a further level of discrimination after TLC. This type of logic shows how the methods complement each other. With the increase in the number of samples, the discriminating power also increases, e.g., in blue fountain pen inks (total 06 samples) DP for four nondiscriminating pairs becomes 0.73 and in case of green fountain pen inks (total 04 samples) with three nondiscriminating pair DP becomes 0.50.

Further study for determination of metal content in blue, black, green, and red fountain pen inks is recommended for their better discrimination.

## Figures and Tables

**Table 1:** Sample of fountain pen inks of different colors and brands.

**Sr. No.**	**Blue**		**Black**		**Green**		**Red**	
	**Ink Brand**	**Sample Code**	**Ink Brand**	**Sample Code**	**Ink Brand**	**Sample Code**	**Ink Brand**	**Sample Code**
1	Dollar (Blue)	BL1	Sheaffer (Black)	BK1	Pelikan (Turquoise)	GN1	Dollar(Red)	RD1

2	Decent (Blue)	BL2	Parker Quink (Black)	BK2	Pelikan (Green)	GN2	Nafees(Red)	RD2

3	Pelikan (Blue)	BL3	Pelikan (Black)	BK3	Parker Quink (Green)	GN3	-	-

4	Deer(Blue)	BL4	Dollar (Black)	BK4	Dollar (Green)	GN4	-	-

5	Piano(Blue)	BL5	Deer(Black)	BK5	-	-	-	-

6	Nafees(Blue)	BL6	Nafees(Black)	BK6	-	-	-	-

**Table 2:** Different absorption bands for blue, black, green and red fountain pen inks using UV/Visible spectroscopy.

**Blue**						**Black**						**Green**				**Red**	
**BL1**	**BL2**	**BL3**	**BL4**	**BL5**	**BL6**	**BK1**	**BK2**	**BK3**	**BK4**	**BK5**	**BK6**	**GN1**	**GN2**	**GN3**	**GN4**	**RD1**	**RD2**
**(nm)**	**(nm)**	**(nm)**	**(nm)**	**(nm)**	**(nm)**	**(nm)**	**(nm)**	**(nm)**	**(nm)**	**(nm)**	**(nm)**	**(nm)**	**(nm)**	**(nm)**	**(nm)**	**(nm)**	**(nm)**
280	280	-	280	-	280	-	235	-	-	-	235	255	-	255	235	-	250

315	315	315	315	315	315	335	315	320	310	305	302	-	-	-	265	-	270

385	-	385	385	-	385	415	-	415	-	-	-	310	305	310	-	330	335

595	605	595	595	601	585	-	-	-	500	510	507	410	385	410	430	505	505

-	-	-	-	-	-	575	590	570	610	595	605	630	640	630	640	-	-

**Table 3:** Discriminating power for blue, black, green and red inks using UV/Visible spectroscopy.

Color of Fountain Pen ink	n= total no. of samples	Total no. of pairs= n(n-1)/2	Discriminating pairs (total no.)	Non Discriminating pairs (total no.)	D.P=no. of discriminating pairs/ Total no. of pairs
Blue	6	6*∗*5/2=15	BL1,BL2;BL1,BL3;BL1,BL5;BL2,BL3;BL2,BL4;BL2,BL5; BL2,BL6;BL3,BL4;BL3,BL6;BL4,BL5;BL5,BL6 (11)	BL1,BL4;BL1,BL6;BL3,BL5; BL4,BL6 (4)	11/15=0.73

Black	6	6*∗*5/2=15	BK1,BK2;BK1,BK4;BK1,BK5;BK1,BK6;BK2,BK3;BK2,BK4; BK2,BK5;BK2,BK6;BK3,BK4;BK3,BK5;BK3,BK6; BK4, BK6; BK5,BK6 (13)	BK1,BK3;BK4,BK5 (2)	13/15=0.87

Green	4	4*∗*3/2=6	GN1,GN2;GN1,GN4;GN2,GN3;GN2,GN4;GN3,GN4 (5)	GN1,GN3 (1)	5/6=0.83

Red	2	2*∗*1/2=1	RD1,RD2 (1)	None (0)	1/1=1

**Table 4:** Discriminating power of blue, black, green and red fountain pen inks using TLC.

Color of Fountain Pen ink	n= total no. of samples	Total no. of pairs= n(n-1)/2	Discriminating pairs (total no.)	Non Discriminating pairs (total no.)	D.P=no. of discriminating pairs/ Total no. of pairs
Blue	6	6*∗*5/2=15	BL1,BL2; BL1,BL4; BL1,BL6;BL2,BL3;BL2,BL4;BL2,BL5;BL2,BL6;BL3,BL4; BL3,BL6;BL4,BL5;BL4,BL6;BL5,BL6 (12)	BL1, BL3; BL1,BL5; BL3,BL5 (3)	12/15=0.80

Black	6	6*∗*5/2=15	BK1,BK2;BK1,BK3;BK1,BK4; BK1,BK5;BK1,BK6;BK2,BK3;BK2,BK4;BK2,BK5;BK2, BK6;BK3,BK4;BK3,BK5;BK3,BK6;BK4,BK5;BK4,BK6; BK5,BK6 (15)	None (0)	15/15=1

Green	4	4*∗*3/2=6	GN1,GN2;GN1,GN3;GN1,GN4;GN2,GN3;GN2,GN4;GN3, GN4 (6)	None (0)	6/6=1

Red	2	2*∗*1/2=1	RD1,RD2 (1)	None (0)	1/1=1

**Table 5:** Calculated Rf values for blue, black, green and red inks using TLC.

**Rf Values of Blue fountain pen Ink**				**Rf Values of Black fountain pen Ink**								**Rf Values of Green fountain pen Ink**							**Rf Values of Red fountain pen Ink**				
**Blue Ink**	**Blue band**	**Sky Blue**	**Purple**	**Black Ink**	**Red**	**Light blue**	**Yellow**	**Orange**	**Light Brown**	**Dark purple**	**Light purple**	**Green Ink**	**Blue**	**Purple**	**Turquoise Green**	**Yellow**	**Sky blue**	**Light green**	**Red Ink**	**Red**	**Dark Pink**	**Yellow**	**Light yellow**
BL1	0.03	0.19	0.34	BK1	0.08	-	0.15	-	0.30	-	-	GN1	0.19	0.37	-	-	0.4	-	RD1	0.09	0.2	0.67	0.71
BL2	0.12	0.19	0.34	BK2	-	0.11	0.28	0.19	-	-	-	GN2	-	-	0.30	0.25	0.07	-	RD2	0.11	0.42	0.67	-
BL3	0.03	0.20	0.34	BK3	0.03	-	0.47	-	0.33	-	-	GN3	0.19	0.37	-	0.30	0.37	0.26	-	-	-	-	-
BL4	0.05	0.20	0.34	BK4	0.08	-	-	0.64	-	0.39	-	GN4	-	-	0.30	0.05	-	-	-	-	-	-	-
BL5	0.03	0.20	0.34	BK5	0.08	-	-	0.67	-	0.42	0.22	-	-	-	-	-	-	-	-	-	-	-	-
BL6	0.03	0.14	0.34	BK6	-	-	-	0.69	-	0.42	-	-	-	-	-	-	-	-	-	-	-	-	-

**Table 6:** Discriminating power of blue, black, green and red fountain pen inks using FTIR.

Color of Fountain Pen ink	n= total no. of samples	Total no. of pairs= n(n-1)/2	Discriminating pairs (total no.)	Non Discriminating pairs (total no.)	D.P=no. of discriminating pairs/ Total no. of pairs
Blue	6	6*∗*5/2=15	BL1,BL2;BL1,BL3;BL1,BL6;BL2, BL3;BL2,BL4;BL2,BL5;BL2,BL6;BL3,BL4; BL3,BL5;BL4,BL6; BL5,BL6 (11)	BL1,BL4; BL1,BL5; BL3, BL6; BL4,BL5 (4)	11/15=0.73
Black	6	6*∗*5/2=15	BK1,BK2;BK1,BK3;BK1,BK4;BK1,BK5; BK1,BK6;BK2,BK3;BK2,BK4; BK2,BK5;BK2,BK6;BK3,BK4;BK3,BK5; BK3,BK6;BK5,BK6 (12)	BK4,BK5; BK4,BK6; BK5,BK6 (3)	12/15=0.80
Green	4	4*∗*3/2=6	GN1,GN3; GN2,GN3;GN3,GN4 (3)	GN1,GN2; GN1,GN4; GN2,GN4; (3)	3/6=0.5
Red	2	2*∗*1/2=1	RD1,RD2 (1)	None (0)	1/1=1

## Data Availability

Raw data files of all techniques are available and can be provided on demand.
